# Case Reports That Illustrate the Efficacy of SGLT2 Inhibitors in the Type 1 Diabetic Patient

**DOI:** 10.1155/2015/676191

**Published:** 2015-02-16

**Authors:** David S. H. Bell

**Affiliations:** Southside Endocrinology, 3928 Montclair Road, Suite 130, Mountain Brook, AL 35213, USA

## Abstract

SGLT2 inhibitors are only approved for use in adults with type 2 diabetes. However, because SGLT2 inhibitors have a mechanism of action that does not require the presence of endogenous insulin, these drugs should also be efficacious in type 1 diabetes where endogenous insulin production is greatly reduced or absent. Herein, I present five cases which illustrate the benefits of utilizing an SGLT2 inhibitor with type 1 diabetes. In these cases the use of SGLT2 inhibitors resulted not only in better glycemic control in most patients but also in some patients' less hypoglycemia, weight loss, and decreased doses of insulin. In type 1 diabetes *Candida albicans* vaginitis and balanitis may occur more frequently than in type 2 diabetes. These cases show that a large randomized clinical trial of SGLT2 inhibitors in type 1 diabetes needs to be performed.

## 1. Introduction

While being not approved for use in type 1 diabetes, SGLT2 inhibitors are being utilized in these patients and there is in the literature documentation of efficacy. In a small phase II placebo-controlled trial the SGLT2 inhibitor dapagliflozin improved glycemic control and reduced insulin needs in type 1 diabetic subjects [1]. A proof of concept study showed that the SGLT2 inhibitor empagliflozin when added to insulin therapy improved glycemic control in type 1 diabetic subjects while lowering the insulin dose and body weight as well as the frequency of hypoglycemia [2]. Improvements in blood pressure, renal hyperfiltration, and arterial stiffness in type 1 diabetes patients have also been documented to occur with empagliflozin [3, 4]. Outside these small clinical trials, the efficacy of SGLT2 receptor blockers in the type 1 diabetic patient, especially in the “real world” practice of medicine, has not been documented.

The positive effects of SGLT2 inhibitors in the type 1 diabetic patient are largely due to the ability of these drugs to increase glucose excretion by lowering the renal threshold for glucose excretion to a level that is not lower than 70 mg/dL. Therefore both the postprandial and preprandial glucose levels can be lowered without increasing the risk of hypoglycemia that would have occurred if the glucose lowering was achieved with short-acting insulins [5, 6]. Thus, in the type 1 diabetic patient when an SGLT2 inhibitor is utilized the dose of preprandial short-acting insulin can and should be reduced and on occasion can even be omitted which will reduce the risk of hypoglycemia. Since with an HbA1c below 7.5% the major contributor to the reduction of the HbA1c is the postprandial rather than the preprandial or fasting glucose levels, the greater reduction of postprandial glucose that occurs due to the lower risk of hypoglycemia ought to result in lower HbA1c levels [7]. In addition, a reduction in glycemic variability has the potential to protect against the development of both the microvascular (retinopathy, nephropathy, and neuropathy) and the macrovascular (ischemic heart disease, peripheral vascular disease, and cerebrovascular disease) complications of type 1 diabetes [8, 9].

Unlike the other oral agents utilized in the treatment of diabetes SGLT2 inhibitors are not dependent upon the presence of endogenous insulin to be effective and because of this insulin-independent mode of action, the SGLT2 inhibitors have the potential to be effective in those type 1 diabetic patients who through resistance to the action of exogenous insulin have been unable to obtain adequate glycemic control. In addition, many patients with type 1 diabetes through the achievement of excellent glycemic control will avoid microvascular complications but gain weight and develop insulin resistance and the metabolic syndrome which may increase the risk of macrovascular complications. With SGLT2 receptor blockade the loss of glucose and calories in the urine will result in weight loss, lowering of insulin resistance, and decreased cardiovascular risk. 

Currently three SGLT2 inhibitors are available (canagliflozin, dapagliflozin, and empagliflozin) which in these case reports are considered to be equally effective so that the need to identify the specific SGLT2 inhibitor that was utilized is not documented. To illustrate the benefits of SGLT2 inhibitors in type 1 diabetes I report five subjects with well-documented type 1 diabetes who benefited from the utilization of an SGLT2 inhibitor. The clinical characteristics of these five patients are shown in Table 1 and their outcomes in Table 2. The potential risks involved in utilizing SGLT2 inhibitors in type 1 diabetes include hypoglycemia, hypotension (especially in the patients utilizing diuretics) syncope, and* Candida albicans* vaginitis in the female and balanitis in the uncircumcised male.

## 2. Case Presentations

### 2.1. Case  1

A 33-year-old white female had had type 1 diabetes (GAD positive, C-peptide positive, 1.1 ng/dL) since the age of 17. On basal/bolus therapy of detemir and lispro she had excellent glycemic control (HbA1c 6.6%) before the initiation of an SGLT2 receptor blocker. As a result of her excellent glycemic control she had no microvascular or macrovascular diabetic complications. Since, as occurs in a small percentage of type 1 diabetic patients, she was C-peptide positive (indicating residual endogenous insulin production) it was hypothesized that her glycemic control could be maintained or even improved on the combination of a basal insulin and an SGLT2 inhibitor without utilizing a short-acting insulin as has been documented to occur in patients with type 2 diabetes [6]. If preprandial short-acting insulin could be removed her risk of hypoglycemia which had been a problem (three or four events per week) would be much less. Her preprandial short-acting insulin doses were reduced by two-thirds when the SGLT2 inhibitor was initiated and she was able to gradually decrease and eventually discontinue her preprandial short-acting insulin without hypoglycemic events. Seven weeks after discontinuing her preprandial short-acting insulin her HbA1c remained at 6.6% and she had not had any episodes of hypoglycemia.

### 2.2. Case  2

A thin (BMI 24) 63-year-old white female who had had type 1 diabetes (GAD positive, C-peptide 0.4 ng/mL (normal range above 1 ng/mL)) since the age of 40 had poorly controlled diabetes (HbA1c 9.6%) due to her resistance to utilizing adequate doses of preprandial short-acting insulin due to her fear of hypoglycemia which in the past had been induced with short-acting insulin. She had on multiple occasions been offered CSII (insulin pump therapy) but had always refused for aesthetic and convenience issues. As a result of her long-term poor glycemic control her type 1 diabetes was complicated by symptomatic, distal, symmetrical, and autonomic diabetic polyneuropathy.

Since she did have low but nevertheless detectable endogenous insulin production, it was hypothesized that the utilization of an SGLT2 inhibitor in combination with a basal-bolus insulin regimen would result in improved glycemic control. Following the initiation of an SGLT2 inhibitor she continued her previous noncompliant habit of underdosing or even deliberately omitting her preprandial short-acting insulin. Nevertheless, in spite of this continued underutilization of short-acting insulin with the initiation of an SGLT2 inhibitor, her postprandial glucose levels dropped and she achieved an HbA1c level of 7.8%.

### 2.3. Case  3

A 68-year-old white male had had type 1 diabetes (C-peptide < 0.1 ng/mL, GAD antibody positive) since the age of 38. As a result of chronic poor glycemic control his diabetes was complicated by background diabetic retinopathy, distal, symmetrical, and autonomic polyneuropathy, and ischemic heart disease. His poor glycemic control was not a compliance issue but was due to frequent, severe, and unpredictable hypoglycemia that occurred whenever the dose of insulin was increased which resulted in underdosing of both his long- and short-acting insulins. He had on several occasions refused CSII (insulin pump therapy) and due to an episode of severe hypoglycemia he had been arrested for erratic driving and his driver's license was suspended. He was therefore unwilling to seek or obtain therapeutic HbA1c (below 8%) levels due to his fear of hypoglycemia so that his HbA1c rose to 10.2% in spite of the use of basal/bolus detemir and aspart insulin therapy along with metformin and pioglitazone.

Following the addition of an SGLT2 inhibitor to his regimen he was able to avoid severe hypoglycemia by reducing or even omitting his preprandial short-acting aspart insulin which was only administered if his preprandial glucose was over 200 mg/dL which usually only occurred once daily. Two months after an SGLT2 inhibitor was added to his regimen his HbA1c had dropped to 8.5% and, with the help of continuous glucose monitoring, severe hypoglycemia was avoided and his driver's license reinstated. Therefore, the addition of an SGLT2 inhibitor resulted in better glycemic control without hypoglycemia and restoration of driving privileges.

### 2.4. Case  4

A 44-year-old white male had type 1 diabetes (GAD positive, C-peptide 0.2 ng/mL) since the age of 18. His diabetes had been poorly controlled (HbA1cs between 10% and 12%) and as a result of this chronic poor glycemic control he had the complications of diabetic nephropathy (creatinine 1.9 mg/dL, estimated GFR 42 mls/min, and urine albumin 67 mg/gram creatinine), distal, symmetrical, and autonomic diabetic neuropathy, and proliferative retinopathy which had in the past required panretinal photocoagulation. He was also morbidly obese (BMI 42) which had induced resistance to the action of insulin so that even on a total insulin dose of 3.4 units/kg poor glycemic control had persisted. Metformin could not be utilized because of his impaired renal function and gastrointestinal symptoms and the addition of sulfonylureas and pioglitazone to his insulin regimen had not significantly improved his glycemic control or reduced his insulin needs. He was, because of his renal function, started a small dose of an SGLT2 blocker resulting in a decreased insulin dose (2.2 units/kg) and an HbA1c of 7.8%.

### 2.5. Case  5

A 42-year-old white female had had type 1 diabetes (C-peptide < 0.1 ng/mL, GAD antibody positive) since the age of 14. Her diabetes had always been very well controlled (HbA1c 6.2–6.8%) so that she had not developed any of the microvascular or macrovascular complications of diabetes. However, on basal-bolus insulin therapy due to her aggressive attempts to achieve excellence in diabetic control she had developed hypoglycemia that had become so severe and frequent that at the age of 38 she reluctantly agreed to be treated with continuous subcutaneous insulin infusion therapy (CSII or insulin pump therapy) which resulted in excellent glycemic control (HbA1c 6.8%) that was not complicated by severe or frequent hypoglycemia. However, as a result of this improved glycemic control her weight increased from 80 kg (BMI 27) to 100 kg (BMI 35) and because of this weight gain pramlintide which was gradually increased to 120 mg t.i.d. was added to her regimen. While her weight was reduced to 82 kg (BMI 28) with pramlintide severe and unpredictable hypoglycemic returned because of the early satiety and decreased food intake that typically occurs with pramlintide. Pramlintide was replaced with liraglutide which was slowly increased to 1.8 mg daily. While there were no further severe or frequent hypoglycemic events on liraglutide therapy and her weight increased to 93 kg (BMI 33), with the addition of an SGLT2 inhibitor to her regimen her weight returned to 80 kg (BMI 28) with maintenance of excellent glycemic control (HbA1c 6.6%) which was achieved without the complication of severe or frequent hypoglycemia.

## 3. Discussion

SGLT2 inhibitors lower both preprandial and postprandial glucose levels by blocking the reabsorption of glucose in the proximal renal tubule [6]. The potential benefit of an SGLT2 inhibitor for the type 1 diabetic patient is that the postprandial glucose is lowered without increasing the risk of hypoglycemia. As shown in case 1, if endogenous insulin production is preserved, a situation that occurs only in a small percentage of type 1 diabetic subjects, there is the potential to completely avoid the use of short-acting insulin which will greatly lower the risk of hypoglycemia while at the same time maintaining glycemic control [10]. Even in the absence of endogenous insulin the dose of preprandial fast-acting insulin can be greatly reduced when an SGLT2 inhibitor is utilized. In case 2 a decreased utilization of short-acting insulin resulted in a lower frequency and severity of hypoglycemia which improved glycemic control. In addition, as illustrated in case 3, many type 1 diabetic patients have very poor glycemic control that cannot be improved with standard basal-bolus therapy because when the insulin doses are raised recurrent, severe, and often unrecognized hypoglycemia which is most often due to the short-acting insulin rather than the long-acting insulin occurs. As shown in case 3, the addition of an SGLT2 inhibitor to the basal-bolus insulin regimen resulted in lower doses of preprandial rapid-acting insulin being utilized leading to less hypoglycemia and improved though not ideal glycemic control [11].

SGLT2 inhibitors are the only oral agents that are effective in inducing weight loss. Many type 1 diabetic patients through achieving ideal HbA1c goals avoid the microvascular complications of diabetes (retinopathy, nephropathy, and neuropathy) while at the same time increasing body weight which results not only in resistance to the action of insulin but also in an increased risk of macrovascular complications (coronary artery, cerebrovascular, and peripheral vascular diseases). In addition, in many of these obese type 1 patients increased and even massive doses of insulin fail to overcome the insulin resistance resulting in little or no improvement in glycemic control. While the use of metformin may help reduce the dose of insulin in most cases metformin is not efficacious [12]. Furthermore, high serum insulin levels are associated not only with an increased risk of cardiovascular disease and cardiovascular mortality but also with an increased risk of cancers (particularly breast, prostate, endometrium, and colon) since insulin is a growth factor and higher insulin levels augment the growth of these tumors [13]. Therefore, the availability of a therapy that is not dependent upon the presence of endogenous insulin will not only improve glycemic control but also decrease the need for high dose insulin therapy in the type 1 diabetic patient as shown in case  4.

Finally, SGLT2 inhibitors by inducing glycosuria will increase calorie excretion which may result in weight loss [6]. In case  5, a type 1 diabetic patient who through achieving excellent glycemic control had increased her body weight by 20%, the addition of an SGLT2 inhibitor to her regimen through increasing glycosuria and urinary calorie loss resulted in the achievement of a more healthy body weight.

The most prevalent complication of SGLT2 therapy in both women and uncircumsized males is the development of* Candida albicans* vaginitis/balanitis which is presumed to be caused by increased glycosuria [6]. While in the patients described in this paper urogenital mycotic infections did not occur, my clinical experience has been that the incidence of these mycotic infections is much greater in the type 1 than in the type 2 diabetic patient. This may be due to subtle T-lymphocyte defects that are present in the type 1 patient and not in the type 2 diabetic patient which would explain the observation of a higher incidence of these opportunistic infections in the type 1 diabetic patient [14].

Larger randomized and blinded studies of SGLT2 inhibitors in type 1 diabetic subject are currently in progress. The experience with these patients described in this report and others suggests that the results of these trials will show that SGLT2 inhibitors are just as beneficial, if not more beneficial, in the type 1 than in the type 2 diabetic patient due to lower postprandial glucose levels, less hypoglycemia, weight loss, and the reduced need for preprandial short-acting insulin. However, these prospective studies may potentially also reveal a higher incidence of mycotic infections particularly* Candida albicans* vaginitis in female and balanitis in the uncircumsized male type 1 diabetic patients than that occurring in the type 2 diabetic patient. However, it should be emphasized that in type 1 diabetes the use of an SGLT2 inhibitor is “off-label” and that no conclusions on the efficacy of SGLT2 inhibitors in type 1 diabetes can be based on these cases. On the other hand, these cases argue that there is a need for large scale randomized clinical trials of SGLT2 inhibitors in type 1 diabetes where the benefits and safety issues can be adequately addressed.

## Figures and Tables

**Table 1:** Characteristics of type 1 diabetic patients before SGLT2 therapy.

Patient	Age in years	Gender	Duration of diabetes (years)	eGFR mls/min	Total daily insulin dose and type
1	33	Female	16	97	Detemir 15 units
					Lispro 10 units

2	63	Female	23	64	Glargine 40 units
					Lispro 12 units

3	68	Male	35	65	Detemir 54 units
					Aspart 10 units

4	44	Male	26	42	Glargine 80 units
					Aspart 300 units

5	42	Female	28	84	Aspart 50 units by insulin pump

**Table 2:** Outcomes of SGLT2 therapy in type 1 diabetic patients.

Patient	HbA1c		BMI		Monthly rate of hypoglycemia	
	Before	After	Before	After	Before	After
1	6.6%	6.6%	25	22	4	0
2	9.6%	7.8%	24	22	2	2
3	10.2%	8.5%	24	21	8	2
4	11.6%	7.8%	42	45	0	0
5	6.8%	6.6%	35	28	8	8
